# Supplementary material for: Wild-Mouse-Derived Gut Microbiome Transplantation in Laboratory Mice Partly Alleviates House-Dust-Mite-Induced Allergic Airway Inflammation
Source: Microorganisms. 2024 Dec 4;12(12):2499. doi: 10.3390/microorganisms12122499 (PMC11728220; doi:10.3390/microorganisms12122499)
Supplement: Supplementary file 1 [file microorganisms-12-02499-s001.zip › microorganisms-3354281-supplementary.pdf]

SUPPLEMETARY MATERIAL

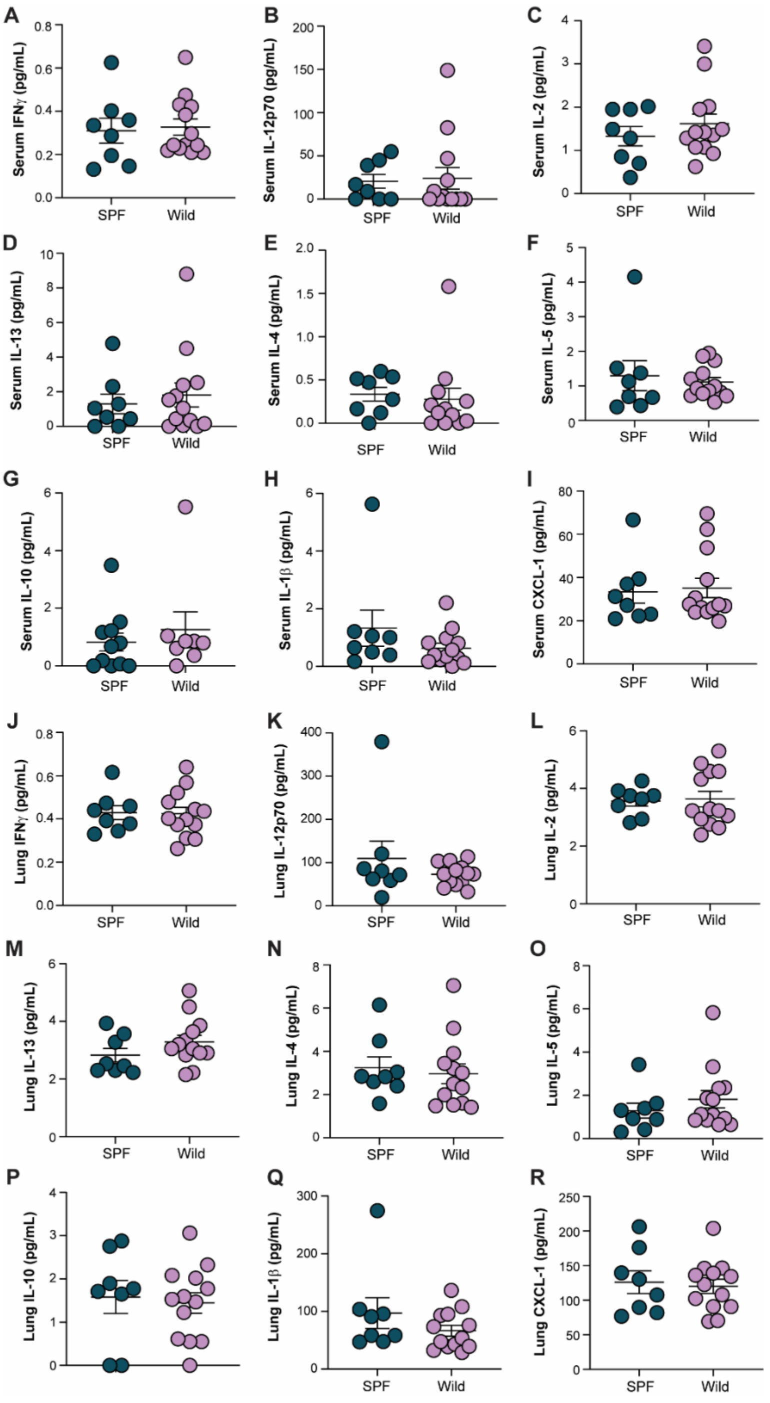

Figure S1. Serum and lung cytokine levels. Concentration of pro-and anti-inflammatory cytokines and chemokines in serum (**A-I**) and inflamed lung tissue (**J-R**) of wild and SPF microbiome associated 7 weeks old male and female BALB/c mice after HDM-induced allergic airway inflammation was induced. Mean and SEM are shown.

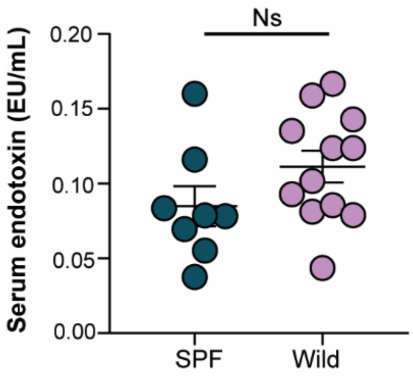

Figure S2. Serum lipopolysaccharide concentration in 7 weeks old SPF and wild microbiome associated mice at euthanization after HDM induction. Mean and SEM are shown. Ns, non-significant.

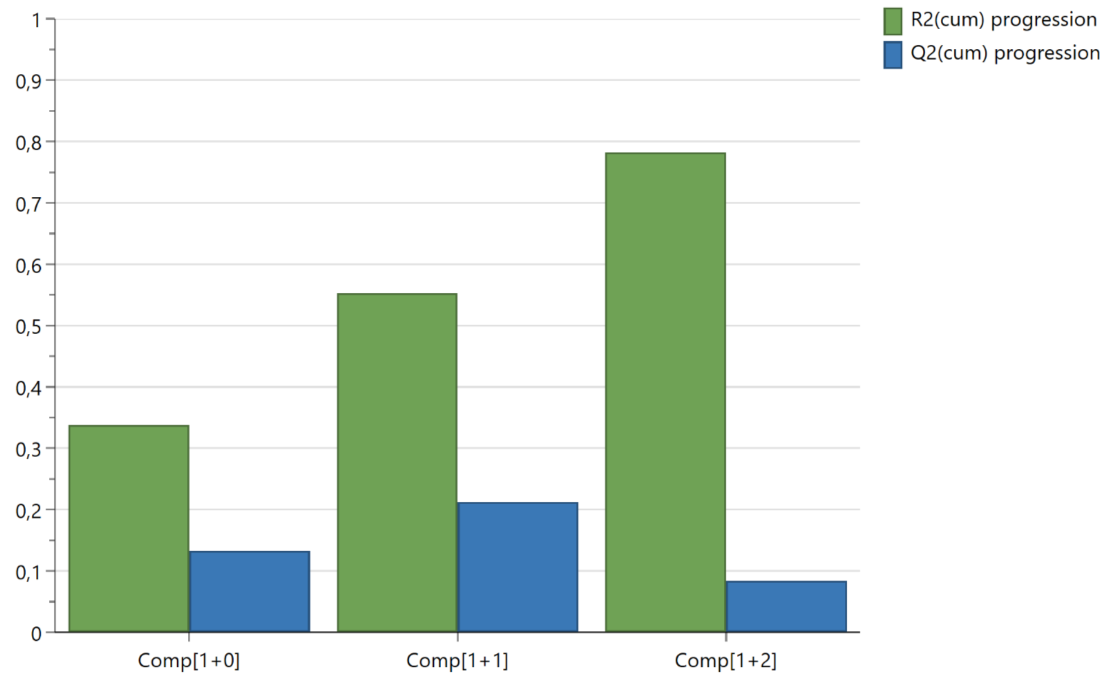

Figure S3. Model overview of the OPLS-DA model for the provided metabolomics dataset. It shows the cumulative R2Y and Q2 coefficients for the groups.

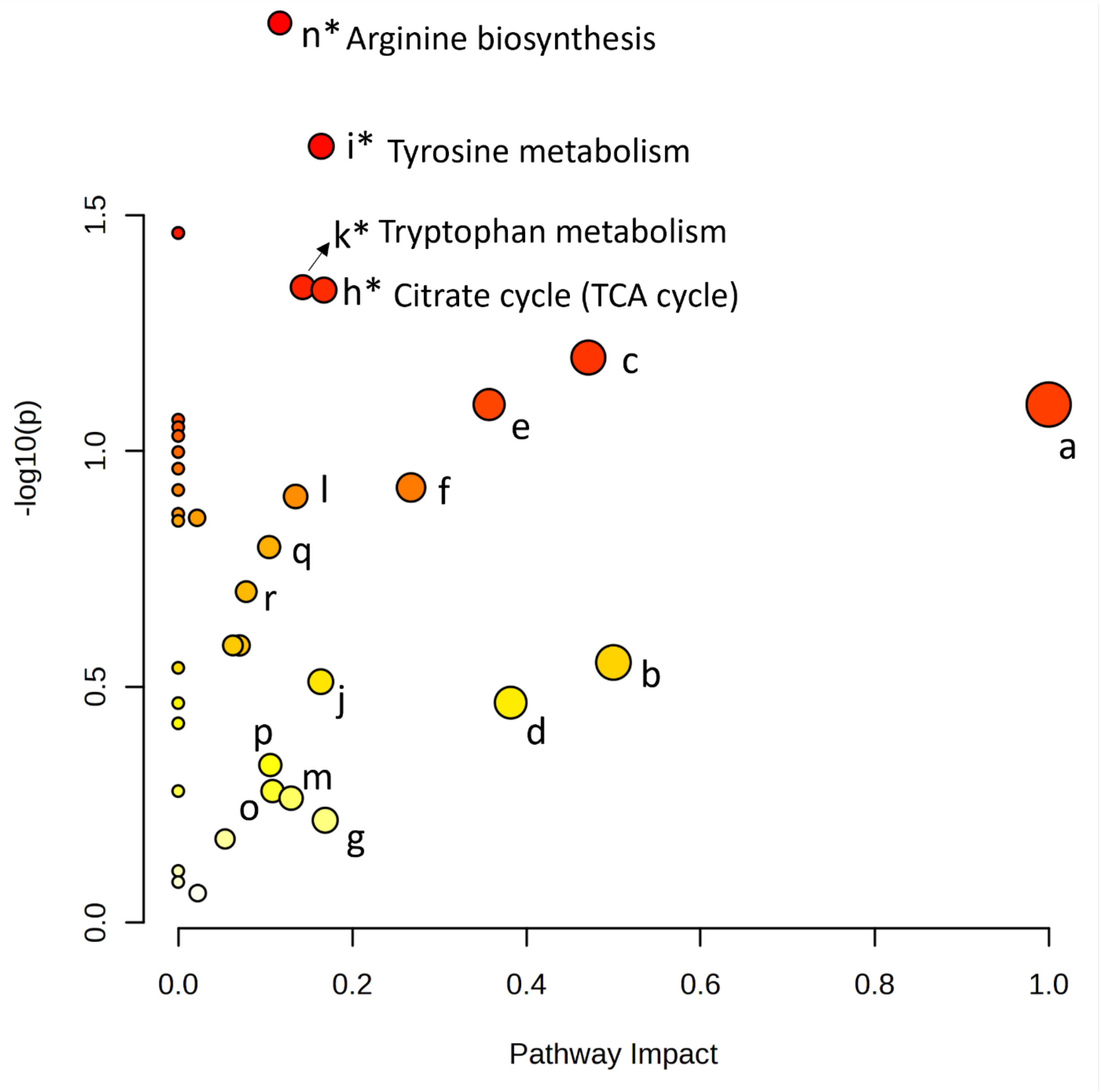

Figure S4. Metabolic pathway analysis. Higher symbols indicate a higher number of metabolites, and red colour indicates high score impact of the respective metabolic pathway. See Tables S2 for complementary information. * Indicate $p < 0.05$ (significant).

Table S1. Cecal metabolite concentrations. P-value < 0.05 indicate significant differences in T tests within each metabolite comparing SPF versus wild animals.

	**Cecal metabolite concentrations (mM)**		
	**Wild**	**SPF**	**p value**
**2'-Deoxyuridine**	0.0052	0.0056	0.7234
**2-Oxoglutarate**	0.0811	0.0531	0.3011
**2-Oxoisocaproate**	0.0341	0.0298	0.5330
**3-Methyl-2-oxovalerate**	0.0349	0.0317	0.9861
**4-Hydroxyphenylacetate**	0.0038	0.0060	0.0125
**Acetate**	1.9130	1.8430	0.7920
**Acetoin**	0.0022	0.0015	0.6311
**Acetone**	0.0021	0.0019	0.8451
**Alanine**	0.0560	0.0705	0.2381
**Aspartate**	0.0245	0.0433	0.0368
**Butyrate**	0.6239	0.5818	0.7070
**Dimethylamine**	0.0005	0.0005	0.6894
**Ethanol**	1.6010	2.4770	0.8044
**Formate**	0.0051	0.0063	0.0721
**Fumarate**	0.0023	0.0007	<0.0001
**Galactose**	0.0365	0.0331	0.5917
**Gallate**	0.0015	0.0030	0.0148
**Glucose**	1.4790	1.5920	0.9718
**Glucose-1-phosphate**	0.0214	0.0293	0.1770
**Glutamate**	0.2083	0.2667	0.1403
**Glycine**	0.0383	0.0390	0.9296
**Hypoxanthine**	0.0994	0.1007	0.9248
**Inosine**	0.0082	0.0057	0.2834
**Isobutyrate**	0.0030	0.0038	0.9577
**Isoleucine**	0.0325	0.0593	0.0535
**Isovalerate**	0.0057	0.0054	0.7100
**Lactate**	0.0109	0.0127	0.2381
**Leucine**	0.0579	0.1027	0.0446
**Lysine**	0.0562	0.1119	0.0126
**Malonate**	0.0092	0.0100	0.4238
**Methanol**	0.0240	0.0231	0.8451
**Methionine**	0.0355	0.0553	0.0430
**Methylamine**	0.0028	0.0025	0.9010
**Nicotinate**	0.0222	0.0233	0.8037
**Phenylalanine**	0.0386	0.0674	0.0126
**Proline**	0.0442	0.0560	0.1042
**Propionate**	0.1827	0.1466	0.1037
**Pyruvate**	0.0180	0.0218	0.1787

**Ribose**	0.2394	0.2492	0.8159
**Sarcosine**	0.0015	0.0020	0.9295
**Succinate**	0.0100	0.0072	0.3270
**Threonine**	0.0550	0.0875	0.0302
**Thymine**	0.0265	0.0219	0.3285
**Trimethylamine**	0.0052	0.0063	0.4024
**Tryptophan**	0.0064	0.0107	0.0287
**Tyrosine**	0.0630	0.0952	0.0246
**Uracil**	0.1240	0.1182	0.7355
**Valerate**	0.0232	0.0184	0.0783
**Valine**	0.0607	0.1073	0.0302
**Xanthine**	0.1063	0.0982	0.8044
**Xylose**	0.0996	0.1164	0.9718
	**Cecal pH**		
**pH**	7.4620	7.5190	0.7404

Table S2. Complete metabolic pathway (Metabolomics data = SPF versus wild).

ID	Pathway	Total	Hits	Raw p	-LOG10 (p)	Holm adjust	FDR	Impact
**a**	Phenylalanine, tyrosine and tryptophan biosynthesis	4	2	0.079753	1.0983	1	0.27432	1
**b**	D-Glutamine and D-glutamate metabolism	6	2	0.28087	0.5515	1	0.43251	0.5
**c**	Alanine, aspartate and glutamate metabolism	28	7	0.0634	1.1979	1	0.27432	0.47116
**d**	Glycine, serine and threonine metabolism	34	4	0.34173	0.46631	1	0.46059	0.38193
**e**	Phenylalanine metabolism	12	2	0.079753	1.0983	1	0.27432	0.35714
**f**	Pyruvate metabolism	22	4	0.11957	0.92237	1	0.27432	0.26749
**g**	Pyrimidine metabolism	39	3	0.60672	0.21701	1	0.67606	0.16886
**h**	Citrate cycle (TCA cycle)	20	4	0.045607	1.341	1	0.27432	0.16744
**i**	Tyrosine metabolism	42	4	0.022603	1.6458	0.85892	0.27432	0.16435
**j**	Arginine and proline metabolism	38	3	0.30853	0.5107	1	0.44566	0.1638
**k**	Tryptophan metabolism	41	1	0.044963	1.3471	1	0.27432	0.14305
**l**	Starch and sucrose metabolism	18	1	0.12493	0.90334	1	0.27432	0.13486
**m**	Glycolysis/Gluconeogenesis	26	6	0.54478	0.26378	1	0.62489	0.12971
**n**	Arginine biosynthesis	14	4	0.012383	1.9072	0.48294	0.27432	0.11675
**o**	Glutathione metabolism	28	2	0.52627	0.27879	1	0.62196	0.10839
**p**	Glyoxylate and dicarboxylate metabolism	32	5	0.46346	0.33399	1	0.58306	0.10582
**q**	Cysteine and methionine metabolism	33	2	0.15992	0.79609	1	0.297	0.10446
**r**	Pentose and glucuronate interconversions	18	2	0.1988	0.70159	1	0.35241	0.07812
**s**	Amino sugar and nucleotide sugar metabolism	37	2	0.25845	0.58762	1	0.41998	0.07059
**t**	Galactose metabolism	27	2	0.25845	0.58762	1	0.41998	0.06283
**u**	Purine metabolism	66	3	0.66484	0.17729	1	0.72024	0.05382
**v**	Primary bile acid biosynthesis	46	1	0.86608	0.06244	1	0.86608	0.02239

**x**	Valine, leucine and isoleucine degradation	40	5	0.13866	0.85805	1	0.27432	0.02168
**y**	Lysine degradation	25	1	0.034513	1.462	1	0.27432	0
**z**	Biotin metabolism	10	1	0.034513	1.462	1	0.27432	0
**aa**	Pantothenate and CoA biosynthesis	19	3	0.08587	1.0662	1	0.27432	0
**ab**	Propanoate metabolism	23	2	0.089034	1.0504	1	0.27432	0
**ac**	Aminoacyl-tRNA biosynthesis	48	14	0.092948	1.0318	1	0.27432	0
**ad**	Histidine metabolism	16	2	0.10054	0.99764	1	0.27432	0
**ae**	Ubiquinone and other terpenoid-quinone biosynthesis	9	1	0.10907	0.96231	1	0.27432	0
**af**	Valine, leucine and isoleucine biosynthesis	8	6	0.12104	0.91706	1	0.27432	0
**ag**	beta-Alanine metabolism	21	2	0.13596	0.8666	1	0.27432	0
**ah**	Nicotinate and nicotinamide metabolism	15	2	0.14068	0.85177	1	0.27432	0
**ai**	Nitrogen metabolism	6	1	0.28834	0.5401	1	0.43251	0
**aj**	Butanoate metabolism	15	4	0.34249	0.46535	1	0.46059	0
**ak**	Selenocompound metabolism	20	1	0.37804	0.42246	1	0.49146	0
**al**	Porphyrin and chlorophyll metabolism	30	2	0.52627	0.27879	1	0.62196	0
**am**	Fatty acid biosynthesis	47	1	0.77725	0.10944	1	0.81926	0
